# Supplementary material for: 1H NMR-Based Metabolomics Reveals the Intrinsic Interaction of Age, Plasma Signature Metabolites, and Nutrient Intake in the Longevity Population in Guangxi, China
Source: Nutrients. 2022 Jun 18;14(12):2539. doi: 10.3390/nu14122539 (PMC9227029; doi:10.3390/nu14122539)
Supplement: Supplementary file 1 [file nutrients-14-02539-s001.zip › nutrients-1762466-supplementary.pdf]

Table S1. Assignments of plasma metabolites

Number	Metabolites	Assignment	δ [1]H(ppm) and multiplicity*
1	Lipids(mainly VLDL)	CH_3, $(CH_2)n$	0.88(t), 1.29(m)
2	Isoleucine	δCH_3, ßCH_3,	0.94(t),1.01(d)
3	Proline	γCH_2	1.99(m)
4	Leucine	δCH_3, ßCH_3	0.95(d)，0.96(d)
5	Valine	γCH_3，γCH_3，αCH	0.99(d)，1.03(d)，3.60(d)
6	Lactate	βCH_3，αCH	1.32(d)，4.11(q)
7	Alanine	βCH_3, αCH	1.47(d), 3.78(q)
8	Acetic Acid	γCH_3	1.91(s)
9	N-acetyl glycoprotein (NAG)	CH_3	2.03(s)
10	Glutamic Acid	βCH_2，γCH_2	2.10(m)，2.34(m)
11	Glutamine	βCH_2, γCH_2	2.12(m), 2.43(m)
12	Propanone	CH_3	2.22(s)
13	Acetoacetic Acid	CH_3	2.27(s)
14	Pyranic Acid	CH_3	2.37(s)
15	Succinic Acid	CH_2	2.41(s)
16	Citrate	CH_2(1/2)，CH_2(1/2)	2.53(d)，2.69(d)
17	Trimethylamine	CH_3	2.86(s)
18	Lysine	βCH	3.03(m)
19	Choline	$N(CH_3)_3$，N-CH_2	3.20(s)，3.66(m)
20	Carnitine	CH_2(COO)	3.21(s)
21	Phosphorylcholine	$N(CH_3)_3$	3.22(s)
22	Trimethylamine oxide (TMAO)	N-(CH3)3)	3.26(s)
23	Scyllitol	CH	3.35(s)
24	β-Glucose	C-H_2，H3/H5，CH_2-C6(1/2)，CH_2-C6(1/2)，CH	3.24(dd)，3.46(m)，3.76(dd)，3.90(dd)，4.64(d)
25	α-Glucose	H4，H2，H3,5-CH，1/2 6-CH_2，1-CH	3.41(m)，.3.53(dd)，3.71(m)，3.83(m)，3.85(m)，5.23(d)
26	Glycine	CH_2	3.56(s)

Number	Metabolites	Assignment	δ [1]H(ppm) and multiplicity*
27	Threonine	αCH	3.57(d)
28	Inositol	4,6-CH	3.63(s)
29	Glycerophosphocholine (GPC)	N-$(CH_3)_3$, NCH_2, P-CH_2	3.61(t),3.91(m), 4.32(t)
30	1-Methylhistidine	αCH，βCH_2(1/2)，βCH_2(1/2)	3.96(dd)，3.07(d)，3.13(d)
31	Creatine	N-CH_3, CH_2	3.03(s), 3.93(s)
32	Triglyceride	$(CH_2)_n$	4.05(m)
33	Unsaturated Lipid	CH=CH	5.29(m)
34	Tyrosine	H2/H6，H3/H5，βCH_2(1/2)，βCH_2(1/2)	7.18(m)，6.88(d)，3.14(m)，3.05(m)
35	Histidine.	2-CH，4-CH	7.02(s)，7.72(s)

* s: single; d: doublet; t: triplet; q: quartet; m: multiplet; dd: doublet of doublet

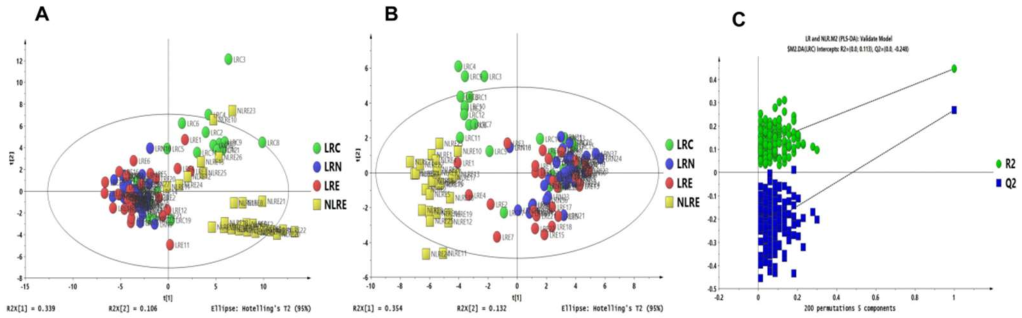

Figure S1. Multivariate analyses of ALL participants. Green circle: LRC, blue circle: LRN, red circle: LRC, and yellow square:NLRE. (A) Scores plot of the PCA model. (B) Score plot of the PLS-DA model. (C) The permutation test result for the PLS-DA model (R2 = (0.0, 0.113), Q2 = (0.0, -0.248)).

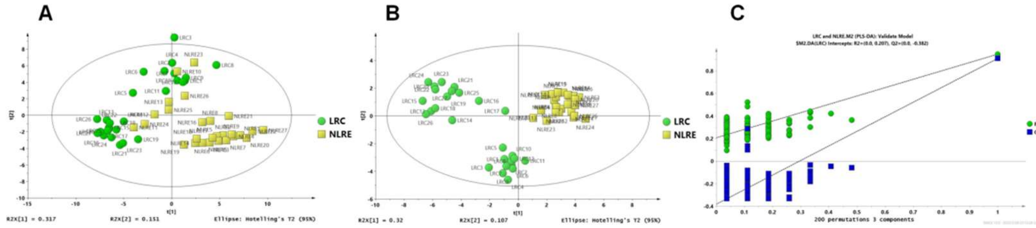

Figure S2. Multivariate analyses of LRC and NLRE. Green circle: LRC and yellow square:NLRE. (A) Scores plot of the PCA model. (B) Score plot of the PLS-DA model. (C) The permutation test result for the PLS-DA model (R2 = (0.0, 0.207), Q2 = (0.0, -0.382)).

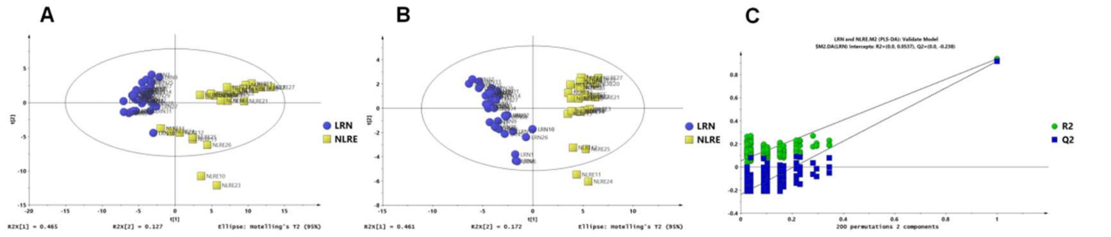

Figure S3. Multivariate analyses of LRN and NLRE. Blue circle: LRN and yellow square:NLRE. (A) Scores plot of the PCA model. (B) Score plot of the PLS-DA model. (C) The permutation test result for the PLS-DA model (R2 = (0.0, 0.0537), Q2 = (0.0, -0.238)).

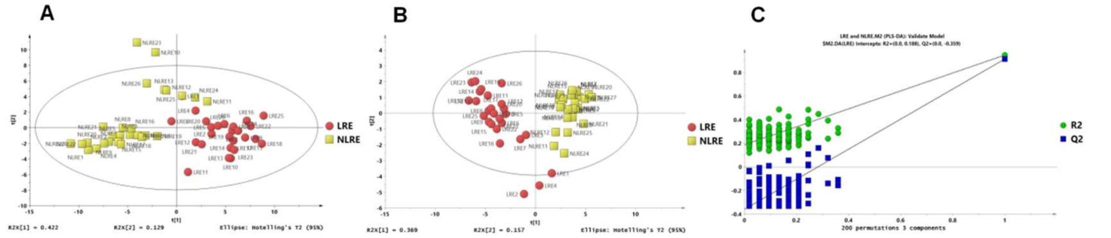

Figure S4. Multivariate analyses of LRN and NLRE. Blue circle: LRN and yellow square:NLRE. (A) Scores plot of the PCA model. (B) Score plot of the PLS-DA model. (C) The permutation test result for the PLS-DA model (R2 = (0.0, 0.188), Q2 = (0.0, -0.359)).

Metabolite Set	Total	Hits	Expect	P value	Holm P	FDR	Details
Glycolysis / Gluconeogenesis	26	3	0.222	0.00113	0.095	0.095	View
Aminoacyl-tRNA biosynthesis	48	3	0.41	0.00674	0.56	0.283	View
Alanine, aspartate and glutamate metabolism	28	2	0.239	0.0225	1.0	0.629	View
Phenylalanine, tyrosine and tryptophan biosynthesis	4	1	0.0342	0.0338	1.0	0.709	View
Valine, leucine and isoleucine biosynthesis	8	1	0.0683	0.0665	1.0	0.989	View
Ubiquinone and other terpenoid-quinone biosynthesis	9	1	0.0769	0.0745	1.0	0.989	View
Phenylalanine metabolism	10	1	0.0854	0.0824	1.0	0.989	View
Glycerolipid metabolism	16	1	0.137	0.129	1.0	1.0	View
Pantothenate and CoA biosynthesis	19	1	0.162	0.151	1.0	1.0	View
Citrate cycle (TCA cycle)	20	1	0.171	0.159	1.0	1.0	View
Fructose and mannose metabolism	20	1	0.171	0.159	1.0	1.0	View
Selenocompound metabolism	20	1	0.171	0.159	1.0	1.0	View
Pyruvate metabolism	22	1	0.188	0.173	1.0	1.0	View
Lysine degradation	25	1	0.214	0.194	1.0	1.0	View
Galactose metabolism	27	1	0.231	0.208	1.0	1.0	View
Glyoxylate and dicarboxylate metabolism	32	1	0.273	0.242	1.0	1.0	View
Glycine, serine and threonine metabolism	33	1	0.282	0.249	1.0	1.0	View
Glycerophospholipid metabolism	36	1	0.307	0.268	1.0	1.0	View
Amino sugar and nucleotide sugar metabolism	37	1	0.316	0.275	1.0	1.0	View
Fatty acid degradation	39	1	0.333	0.287	1.0	1.0	View
Valine, leucine and isoleucine degradation	40	1	0.342	0.294	1.0	1.0	View
N-Glycan biosynthesis	41	1	0.35	0.3	1.0	1.0	View
Tyrosine metabolism	42	1	0.359	0.306	1.0	1.0	View

Figure S5. Metabolic Pathway enrichment of deregulated metabolites involved in LRC, LRN, LRE and NLRE

Table S2. Detailed information of all participants

Number	Gender	Age(years)	BMI (kg/m^2)
NLRE1	female	60	21.48
NLRE2	male	61	24.84
NLRE3	female	61	22.48
NLRE4	male	63	25.39
NLRE5	female	63	22.43
NLRE6	male	64	25.74
NLRE7	male	65	21.78
NLRE8	female	66	24.65
NLRE9	male	66	21.09
NLRE10	female	69	25.97
NLRE11	female	70	20.73
NLRE12	female	70	20.07
NLRE13	male	70	25.77
NLRE14	male	71	25.62
NLRE15	female	71	25.15
NLRE16	male	72	18.24
NLRE17	male	72	22.96
NLRE18	female	73	26.27
NLRE19	female	74	19.91
NLRE20	male	75	22.10
NLRE21	male	78	24.01
NLRE22	male	81	18.63
NLRE23	male	81	33.18
NLRE24	female	82	26.27
NLRE25	female	83	21.55
NLRE26	female	83	23.73
NLRE27	female	86	17.15
LRE1	male	60	18.59
LRE2	male	61	18.36
LRE3	male	62	24.74
LRE4	male	62	27.18
LRE5	male	63	17.27
LRE6	male	63	23.34
LRE7	female	64	22.43
LRE8	female	64	22.03
LRE9	female	65	18.67
LRE10	male	65	23.69
LRE11	male	66	23.20
LRE12	male	66	24.01
LRE13	female	67	18.11

Number	Gender	Age(years)	BMI (kg/m^2)
LRE14	male	68	20.32
LRE15	male	68	25.06
LRE16	male	69	27.04
LRE17	male	70	27.48
LRE18	male	71	24.58
LRE19	male	73	29.04
LRE20	male	75	29.00
LRE21	female	77	19.73
LRE22	female	79	24.12
LRE23	female	82	21.21
LRE24	female	84	15.36
LRE25	female	87	19.81
LRE26	male	89	16.17
LRN1	female	90	12.42
LRN2	male	91	20.08
LRN3	female	91	18.11
LRN4	female	91	22.58
LRN5	female	91	19.56
LRN6	female	91	19.17
LRN7	female	91	27.77
LRN8	female	91	21.39
LRN9	female	91	14.97
LRN10	male	91	18.09
LRN11	male	91	20.02
LRN12	female	92	14.74
LRN13	female	92	20.48
LRN14	female	92	19.50
LRN15	female	92	14.60
LRN16	female	92	18.69
LRN17	female	92	19.44
LRN18	female	92	20.41
LRN19	female	92	20.10
LRN20	male	92	15.12
LRN21	female	93	18.07
LRN22	female	93	19.82
LRN23	female	93	17.33
LRN24	female	93	17.22
LRN25	male	93	17.64
LRN26	male	94	20.60
LRN27	male	94	28.84
LRN28	male	94	21.99
LRN29	female	95	18.38

Number	Gender	Age(years)	BMI (kg/m^2)
LRN30	female	95	19.70
LRN31	female	95	22.74
LRN32	female	95	14.91
LRN33	female	96	19.94
LRN34	female	96	14.96
LRN35	female	98	17.58
LRN36	female	98	18.92
LRN37	female	98	17.36
LRC1	male	100	12.42
LRC2	female	100	20.08
LRC3	female	100	18.11
LRC4	male	100	22.58
LRC5	female	100	19.56
LRC6	female	101	19.17
LRC7	female	101	27.77
LRC8	female	101	21.39
LRC9	male	101	14.97
LRC10	female	101	18.09
LRC11	female	101	20.02
LRC12	female	102	14.74
LRC13	female	102	20.48
LRC14	female	102	19.50
LRC15	female	102	14.60
LRC16	female	102	18.69
LRC17	female	102	19.44
LRC18	female	102	20.41
LRC19	female	104	20.10
LRC20	female	104	15.12
LRC21	female	106	18.07
LRC22	female	106	19.82
LRC23	female	107	17.33
LRC24	female	108	17.22
LRC25	female	108	17.64
LRC26	female	111	20.60
LRC27	female	118	28.84
